# Physicians' Motives for Professional Internet Use and Differences in Attitudes Toward the Internet-Informed Patient, Physician–Patient Communication, and Prescribing Behavior

**DOI:** 10.2196/med20.1996

**Published:** 2012-07-06

**Authors:** Martina Moick, Ralf Terlutter

**Affiliations:** ^1^Department of Marketing and International ManagementSchool of Management and EconomicsAlpen-Adria-Universitaet KlagenfurtKlagenfurtAustria

**Keywords:** Physician, Internet use, attitude, Internet-informed patient, communication, prescribing behavior, physician-patient relationship, motivation research

## Abstract

**Background:**

Physicians have differing motives for using the Internet and Internet-related services in their professional work. These motives may affect their evaluation of patients who bring with them health-related information from the Internet. Differing motives may also affect physician–patient communication and subsequent prescribing behavior.

**Objectives:**

To segment physicians into types based on their motives for using the Internet in connection with professional activities and to analyze how those segments differ in their attitudes in three areas: toward patients who bring along Internet-sourced information; in their own subsequent prescribing behavior; and in their attitudes toward using the Internet to communicate with patients in future.

**Methods:**

We surveyed 287 German physicians online from three medical fields. To assess physicians’ motives for using the Internet for their professional activities, we asked them to rate their level of agreement with statements on a 7-point scale. Motive statements were reduced to motive dimensions using principal component analysis, and 2-step cluster analysis based on motive dimensions identified different segments of physicians. Several statements assessed agreement or disagreement on a 7-point scale physicians’ attitudes toward patients’ bringing Internet information to the consultation and their own subsequent prescribing behavior. Further, we asked physicians to indicate on a 7-point scale their valuation of the Internet for physician–patient communication in the future. Data were then subjected to variance and contingency analyses.

**Results:**

We identified three motive dimensions for Internet use: (1) being on the cutting edge and for self-expression (Cronbach alpha = .88), (2) efficiency and effectiveness (alpha = .79), and (3) diversity and convenience (alpha = .71). These three factors accounted for 71.4% of the variance. Based on physicians’ motives for using the Internet, four types of physician Internet user were identified: (1) the Internet Advocate (2), Efficiency-Oriented, (3) Internet Critic, and (4) Driven Self-expressionist. Groups differed significantly concerning (1) their attitude toward informed patients in general (*F*_1234 _= 9.215, *P *< .001), (2) perceived improvement in the physician–patient relationship Internet information brings (*F*_1234 _= 5.386, *P *< .001), (3) perceived accuracy of information the patient brings (*F*_1234 _= 3.658, *P *= .01), and (4) perceived amount of time needed to devote to an Internet-informed patient (*F*_1234 _= 3.356, *P *= .02). Physician segments did not differ significantly in reported prescribing behavior (*F*_1234 _= 1.910, *P *= .13). However, attitudes toward using the Internet to communicate with patients in future differed significantly (*F*_1234 _= 23.242, *P *< .001).

**Conclusions:**

Based on self-reporting by German physicians of their motives for professional Internet use, we identified four types of Internet users who differ significantly in their attitude toward patients who bring along Internet information and their attitudes toward using the Internet to communicate with patients in future.

## Introduction

The Internet has become an important tool for finding medical information and in medical care. Increasingly physicians are using Internet services in their professional work. However, little is known about the motives behind physicians’ use of Internet services. In this research, we analyzed physicians’ motives for professional Internet use. We segmented physicians based on their motives for professional Internet use and analyzed how the segments differ with regard to their attitude toward patients who bring information taken from the Internet to a consultation with their doctor, physicians’ related prescribing behavior, and their attitudes toward the possibilities for Internet communication with patients in the future. Previous surveys have particularly concentrated on physicians’ use of specific media channels (eg, email) [1], their information seeking on the Internet, and the impact of media on medical education [2,3]. Surveys have also been conducted on the physician–patient relationship, but primarily from the patient’s perspective [4-7]. As previous research has shown that the correlation of Internet affinity and Internet usage of physicians based on general demographic data such as age is continuously decreasing [8-10], we argue that motive research might prove a useful technique for increasing our understanding of physicians’ professional Internet use and related variables.

The paper addresses the following research questions: (1) What different types do physicians fall into based on their motives for using the Internet for professional activities? (2) How do segments differ with regard to their evaluation of patients who bring Internet information to a consultation? (3) How do physicians differ with regard to the pressure they feel to prescribe a requested pharmaceutical when a patient brings along information from the Internet? (4) How do they differ with regard to their attitude toward using the Internet to communicate with patients in future?

### Physicians’ Motives for Professional Internet Use

In our study, we segmented physicians based on their motives for professional Internet use. Motives are strong driving forces of human behavior [11]. They are goal oriented and make individuals engage in actions to accomplish their goals [12]. We expect that physicians are likely to differ in their motives for engaging in Internet-related activities in their professional work. For instance, whereas one physician may engage in Internet activities only to avoid giving the impression of lagging behind colleagues, another physician may see true advantages in health-related Internet services. While one physician may use the Internet primarily to seek information, another physician’s motivation may be more related to social aspects of health communities. We argue that physicians can probably be segmented based on their motives for professional Internet use and that those segments probably differ with regard to their evaluation of patients who bring health-related information to a consultation, their subsequent prescribing behavior with those patients, and their attitude toward using the Internet for communication with patients in future. In consumer and marketing research, motives are regularly used as segmentation variables to predict attitudes and behavior [11,12]. We selected the motives used in this study by considering different aspects of Internet use. They were initially derived from literature and then further developed in consultation with two medical experts (Multimedia Appendix 1 lists all items used in the item set, including the origin sources). We especially focused on items that deal with the typical characteristics of Internet use (eg, time factors and ease of use) [2,13-16], professional activities [2,17], interaction through the Internet, and diversity of formats [15,18].

### Dependent Variables

#### Attitude Toward Patients Who Bring Along Health-Related Internet Information

Because the Internet offers broad and easy access to health-related information [2,13], physicians are increasingly confronted with patients who bring along data from the Internet. Over recent years, there has been a shift from passive and uninformed patients to empowered consumers who take better care of their own health [4,19]. However, a patient’s being informed can have a positive or a negative effect on communication and the physician–patient relationship [4,20,21]. On the one hand, informed patients can communicate better, since the Internet improves their understanding of their condition or treatment, and time can be used more efficiently [5,22,23]. In a study among Israeli physicians, about 60% of physicians reported being satisfied with patients who bring data from the Internet to the consultation [24]. On the other hand, since patients have access to a pool of professional as well as lay materials [25], information is often linked with wrong, misleading, or unreliable content, and expert information can be misunderstood by patients [26,27]. Though the findings are ambiguous (eg, [28]), previous research also shows concerns regarding unnecessary consultations [29], time-consuming discussions [22], dealing with questions [4], and correcting misinterpreted information [21], all of which are a challenge to physicians. Previous research has also demonstrated that physicians often lag behind their patients in their Internet usage [26,29] and their knowledge of information technologies [30]. Furthermore, discussions due to misleading interpretations by patients [21] may harm the physician–patient relationship [31]. Physicians encounter patients who expect their physician to interpret the health-related Web content and so may feel challenged and pressured to have a higher level of information [32]. With the shift from the passive patient to the empowered one [4], physicians who dislike having their authority questioned may have a problem with the new patient-initiated collaborative role [24,29]. Thus, the question arises as to how physicians segmented according to their motives for professional Internet use differ in their attitudes to patients who bring along Information from the Internet. In this study, we analyzed attitudes toward the Internet-informed patient in general, as well as physicians’ perceptions of improvement in the physician–patient relationship, the amount of wrong and misunderstood information patients bring, the amount of time consumed in consultations, and the loss of physicians’ authority and control.

#### Prescribing Behavior

With the growing volume of publically available information, patients may want to be increasingly involved in the decision-making process. Results of empirical studies among physicians are mixed. In a study among Greek physicians, results indicate that physicians are still autonomous, and only 11% would prescribe a medication requested by the patient [33]. A study by Richard and Lussier suggested similar results: the physician has the role of provider and the patient that of listener [34]. However, a study among UK physicians found that, in approximately 50% of prescriptions, patients’ preferences were considered [35], because physicians felt pressured by the patients. About 75% of US physicians reported preferring shared decision making with their patients and especially encouraged their patients to look for information [31]. Findings were similar among Swiss physicians, who appreciated Internet-informed patients participating actively in the consultation [21]. The next research question that we addressed was whether the segments of physicians differ in their prescribing behavior if a patient with Internet information requests a specific medication.

#### Attitudes Toward Internet Communication in the Future

The Internet has already become a common tool for physicians’ activities: it is used to seek information, to post content in blogs and on bulletin boards, and to communicate with other medical professionals [17,36]. Empirical studies report the potential for using the Internet for communication with patients, for instance to reduce office visits [37] and to improve chronic disease management [38]. In particular, text-based consultation is expected to increase in the near future [18]. Other studies report positive attitudes from patients and physicians toward online communication [9], although telemedicine consultation seems to be more physician centered [39]. However, a study by Bosslet et al found that about 50% of physicians are pessimistic regarding potential improvements in physician–patient communication, seeing potential threats to patient confidentiality [36]. At least one-third of the overall population in selected European countries was interested in the possibility of using a Web tool to renew prescriptions, schedule appointments, or ask the doctor health questions [40]. The final question that this research addressed was how the segments of physicians differ in their estimation of future Internet communication with patients.

## Methods

We surveyed 287 German physicians in December 2010 and January 2011. The survey contained a set of questions about use of the Internet, attitudes toward Internet-informed patients, prescribing behavior, and attitudes toward Internet communication in the future. The sample was drawn from a physicians’ e-panel maintained by GfK HealthCare, a survey research company in Nuremberg, Germany. The sample was based on a randomly generated set of physicians stratified by medical field, consisting of general practitioners (n = 127), orthopedists (n = 80), and dermatologists (n = 80). The survey was continued until the determined number of participants had taken part (for a detailed summary of the survey, see Multimedia Appendix 2). We chose these particular medical fields because all three types of physician have to treat both acute and chronic diseases, which allowed some comparisons between medical fields. Data were analyzed using SPSS version 18 (IBM Corporation, Somers, NY, USA).

### Physicians’ Motives for Internet Use in Professional Activities

To assess the physicians’ motives for using the Internet for their professional activities, we gave respondents a list of statements relating to their professional work and asked them to state their level of agreement with the statements on a 7-point scale (1, strongly disagree; to 7, strongly agree). For example, one statement was “The Internet offers an opportunity to express oneself” (see Table 1 and Multimedia Appendix 1). To identify the segments according to different user types, we analyzed the data in 2 steps. First, a principal component analysis reduced the data to underlying motive dimensions. Second, we used an exploratory cluster analysis [41] to segment the types of Internet users. We applied 2-step cluster analysis, as we had no expectations regarding the number of clusters. This analysis is a combination of hierarchical clustering and nonhierarchical clustering [41,42]. The analysis was done in 2 steps and is based on Euclidean distance measures. In the first step, cases were preclustered into subclusters using a sequential clustering approach [43]. In the second step, preclusters were analyzed by agglomerative hierarchical clustering. The algorithm for agglomeration is based on the Schwarz Bayesian criterion to evaluate the number of clusters and to refine the initial estimate [44]. By using an analysis of variance, we confirmed that the variables included in the cluster analysis differed significantly in at least two of the variables of the identified clusters (see Table 2 and Multimedia Appendix 3). To get a more detailed description of different types of Internet users, we also described the groups on the basis of demographic data: medical field, age, and sex. Descriptive variables used also included the type of disease treated (chronic vs acute), feelings about the Internet in general, and duration of private and professional use of the Internet (see Table 3).

### Attitude Toward Patients Who Bring Along Health-Related Internet Information

We assessed this by asking about the level of agreement (1, strongly disagree; to 7, strongly agree) with statements concerning the physicians’ general attitude toward these patients; whether the physician expected an improvement in the physician–patient relationship; whether he or she expected wrong or misunderstood information; whether he or she expected a more time-consuming consultation; and whether he or she perceived a loss of authority and control. For example, one statement was “If a patient brings health-related information from the Internet in consultation, I think it is generally positive.” (See Multimedia Appendix 1 for complete wording of statements.)

### Prescribing Behavior

We investigated prescribing behavior by rating the level of agreement (1, strongly disagree; to 7, strongly agree) with the statement “If a patient brought some health-related information to the consultation, I would be more likely to prescribe a desired medication than if the patient was uninformed.”

### Attitudes Toward Internet Communication in the Future

We asked “Could you imagine using the Internet for communication with your patients more often in the future?” Responses to this question were rated on a 7-point scale (1, I absolutely cannot imagine; to 7, I can easily imagine).

## Results

### User Types Based on Their Motive for Internet Use

Principal component analysis with varimax rotation revealed that motives had three underlying factors (motive dimensions). The factors were (1) being on the cutting edge and for self-expression (Cronbach alpha = .88), (2) efficiency and effectiveness (alpha = .79), and (3) diversity and convenience (alpha = .71) (Table 1). The three factors accounted for 71.4% of variance. Due to a low factor loading, 4 of 14 statements were not included in any of the factors and we omitted them from further analyses.

On the basis of the motive dimensions for using the Internet for professional activities, we identified four types of Internet user by a 2-step cluster analysis: (1) the Internet Advocate, (2) the Efficiency-Oriented physician, (3) the Internet Critic, and (4) the Driven Self-expressionist (Table 2). Analyses of variance (see Table 3) and contingency analyses revealed differences between the segments (see Multimedia Appendix 3 for details). The four segments differed significantly with regard to medical field (χ^2^_1 _= 16.7, *P *= .01), duration of private Internet use (*F *= 4.173, *P *= .01), duration of professional Internet use (*F *= 3.351, *P *= .02), and feelings about the Internet and Web 2.0 in general (*F *= 7.433, *P *< .001). No significant differences were found with regard to age (χ^2^_1 _= 1.7, *P *= .95), sex (χ^2^_1 _= 4.1, *P *= .26), or type of disease treated (chronic vs acute) (χ^2^_1 _= 6.0, *P *= .42).

**Table 1:** Principal component analysis with varimax rotation of physicians’ motives for using the Internet for professional activities.

Motive dimension		Factor 1	Factor 2	Factor 3
**Factor 1: Cutting edge and self-expression**				
	It is important to be on the Web as a physician	0.892	0.173	0.041
	It offers an opportunity to express oneself	0.890	0.122	0.132
	I want to be on the cutting edge	0.790	0.159	0.220
	I want to keep up with other physicians	0.748	–0.022	0.276
**Factor 2: Efficiency and effectiveness**				
	I can look for information easily	0.061	0.896	0.047
	It offers a vast amount of information	0.053	0.882	0.185
	It offers current information	0.049	0.601	0.506
	I want to save time	0.255	0.581	0.134
**Factor 3: Diversity and convenience**				
	The information is easy to understand	0.188	0.180	0.826
	It offers different formats, eg, social networks, podcasts, or health bulletin boards	0.278	0.164	0.794
Eigenvalue		4.198	1.933	1.005
Variance explained		29.597	24.103	17.665
Cronbach alpha		.88	.79	.71

**Table 2:** Analysis of user segments based on motive dimension, mean of factor values (SD).

Motive dimension	User type				*F*_1234_	*P *value
	Internet Advocate (n = 101)	Efficiency- Oriented (n = 93)	Internet Critic (n = 29)	Driven Self- Expressionist (n = 51)		
Cutting edge and self-expression	0.683 (0.548)	–1.050 (0.508)	–0.261 (0.865)	0.711 (0.646)	166.946	<.001
Efficiency and effectiveness	0.085 (0.556)	0.314 (0.559)	–2.214 (1.158)	0.518 (0.450)	138.868	<.001
Diversity and convenience	0.675 (0.450)	0.000 (1.026)	–0.470 (0.900)	–1.071 (0.667)	61.601	<.001

**Table 3:** Characteristics of user types.

Characteristic		No.	User type				Total	*F/*χ^2^_1234_	*P *value
			Internet Advocate	Efficiency- Oriented	Internet Critic	Driven Self- expressionist			
**Medical field**								16.7	.01
	General practitioner	123	54 (44)	45 (37%)	7 (6%)	17 (14%)	100.0%		
	Orthopedist	77	28 (36%)	18 (24%)	11 (14%)	20 (26%)	100%		
	Dermatologist	74	19 (26%)	30 (40%)	11 (15%)	14 (19%)	100%		
**Sex**								4.1	.26
	Male	231	84 (36%)	78 (34%)	22 (10%)	47 (20%)	100.0%		
	Female	43	17 (40%)	15 (35%3)	7 (16%)	4 (9%)	100%		
**Age range (years)**								1.7	.95
	30–42	37	16 (43%)	10 (27%)	3 (8%)	8 (22%)	100%		
	43–55	169	60 (36%)	60 (36%)	18 (11%)	31 (18%)	100.0%		
	56–64	67	25 (37%)	22 (33%)	8 (12%)	12 (18%)	100%		
**Daily Internet use (hours), mean**									
	Private use	274	1.62	1.15	1.55	1.21	1.38	4.173	.01
	Professional use	274	1.25	0.83	0.87	0.82	0.99	3.351	.02
**Feelings about the Internet and Web 2.0 in general, mean score** **^a^**		270	6.09	5.61	4.85	5.73	5.74	7.433	<.001
**Type of disease treated**								6.0	.42
	Chronic	34	14 (41%)	8 (24%)	4 (11%)	8 (24%)	100%		
	Acute	6	4 (66%)	0 (0%)	1 (17%)	1 (17%)	100%		
	Both equally	234	83 (36%)	85 (36%)	24 (10%)	42 (18%)	100.0%		

^a ^1, strongly disagree; 7, strongly agree.

The *Internet Advocate *(n = 101, 35.2% of sample) is the physician segment with the most positive evaluation of professional Internet use. This type wants to be on the cutting edge and in particular appreciates the diversity of formats of user-generated media, such as social networks and bulletin boards. These physicians find the Internet useful for self-expression and finding information easily. A total of 44% (54/123) of general practitioners were in this group and 36% (28/77) of orthopedists, but only 26% (19/74) of dermatologists. Hence, general practitioners were overrepresented, while dermatologists were underrepresented in this segment. This distribution is probably related to physicians’ target patient groups. General practitioners treat patients from a broader age range than do physicians in other medical fields. Among the four segments, the Internet Advocate had the highest daily usage of the Internet, with an average 1.62 hours for private and 1.25 hours for professional activities. This segment had the most positive feelings about the Internet and Web 2.0 in general (mean score of 6.09 on a 7-point scale).

The *Efficiency-Oriented *physician (n = 93, 32%) appreciates the Internet mainly for its convenience, speed, and ease of finding information. The majority of the dermatologists (30/74, 40%) belonged to this user segment, 37% (45/123) of the general practitioners, and 24% (18/77) of all orthopedists. This type used the Internet the least of all four types, on average 1.15 hours per day for private and 0.83 hours for professional activities. Their feelings about the Internet and Web 2.0 applications were generally positive (mean score of 5.61 on a 7-point scale).

The *Internet Critic *(n = 29, 10%) was the smallest segment. This type had rather low ratings regarding the motives for Internet use. Only about 6% (7/123) of general practitioners, 14% (11/77) of orthopedists, and 15% (11/74) of dermatologists belonged to this group. General practitioners were underrepresented, whereas orthopedists and dermatologists were overrepresented in this segment. Interestingly, the Internet Critic had the second highest rate of Internet use for private activities (mean of 1.55 hours daily), but a much lower use for professional activities (0.87 hours daily). This user type had the least positive feelings regarding the Internet (average score of 4.85). Despite the comparatively high use of the Internet privately, physicians of this type obviously did not see enough advantage in Internet use for professional work. One possible interpretation of the relatively long time spent in private use of the Internet combined with the relatively low evaluation of the Internet in general may be that this segment of physicians is less efficient at using the Internet, such as for finding the relevant information. They may just have a poorer Internet literacy. However, additional research is needed here.

The *Driven Self-expressionist *(n = 51, 18%) uses the Internet for self-expression and sees the importance to a physician of being on the Web. This segment uses the Internet for its convenience, but has low motivation to use user-generated media. Orthopedists (20/77, 26% of all orthopedists) were overrepresented in this group, whereas general practitioners were slightly underrepresented (17/123, 14%). Of all dermatologists, 19% (14/74) were in this segment. The duration of Internet use on average was 1.21 hours a day for private activities and 0.82 hours for professional activities. This user type had positive feelings regarding the Internet (average score of 5.73).

### Attitude Toward Patients Who Bring Along Health-Related Internet Information

Attitudes toward patients who bring information from the Internet to a consultation differ significantly between the four physician groups (see Table 4).

**Table 4:** Analysis of differences between user types based on mean scores^a^.

Attitude		User type				Total	*F_1234_*	*P *value
		Internet Advocate	Efficiency- Oriented	Internet Critic	Driven Self- Expressionist			
**Attitudes toward Internet-informed patients**								
	Positive attitude in general	5.21	4.27	3.90	4.18	4.56	9.215	<.001
	Improvement of physician–patient relationship	4.68	4.05	3.59	4.06	4.24	5.386	<.001
	Wrong and misunderstood information	5.59	5.44	4.97	5.88	5.53	3.658	.01
	Time-consuming consultation	4.97	5.27	4.17	5.12	5.01	3.356	.02
	Loss of authority and control	2.80	2.60	2.83	3.06	2.78	0.879	.45
Attitudes toward prescribing a patient’s desired medication		3.28	2.86	2.66	3.24	3.06	1.910	.13
Attitude toward using the Internet for communicating with patients in future		4.91	3.36	2.18	4.45	4.02	23.242	<.001

^a ^1, strongly disagree; 7, strongly agree.

With regard to physicians’ attitudes toward the information level of patients in general, the Internet Advocate had the most positive attitude, with a mean of 5.21, compared with the Efficiency-Oriented (4.27), the Driven Self-expressionist (4.18), and the Internet Critic (3.90; *F *= 9.215, *P *< .001). The Internet Advocate also saw a high degree of benefit for the physician–patient relationship in enhanced information levels (mean 4.68), while the Driven Self-expressionist (4.06) and the Efficiency-Oriented (4.05) agree, but less positively. The Internet Critic (3.59) saw the least benefit for improving the relationship (*F *= 5.386, *P *< .001). The view that Internet-informed patients often come with wrong, incomplete, or misunderstood information also differed significantly. Whereas the Internet Critic had the least negative opinion here (mean 4.97), the Efficiency-Oriented (5.44), the Internet Advocate (5.59), and the Driven Self-expressionist (5.88; *F *= 3.658, *P *= .01) thought that patients are not able to deal with health-related Internet information correctly or are unable to differentiate between accurate and inaccurate content. Notably, all the mean scores are quite high, indicating that all four physician groups thought that Internet-informed patients are often misinformed. Concerning time-consuming consultations, the Internet Critic was least likely to expect additional communication time (mean 4.17), compared with the Internet Advocate with 4.97, the Driven Self-expressionist with 5.12, and the Efficiency-Oriented with 5.27 (*F *= 3.356, *P *= .02). There were no significant differences between physician groups concerning the loss of power and control (*F *= 0.879, *P *= .45). All user types stated that they did not feel challenged in their authority by patients with Internet information.

### Prescribing Behavior

There were no differences between the four segments with regard to the pressure they felt to prescribe a medication that a patient requests depending on whether the patient is informed (*F *= 1.910, *P *= .13). All four segments had mean scores below the midpoint of the scale (means varied from 2.66 to 3.28) (Table 4).

### Attitudes Toward Internet Communication With Patients in the Future

Attitude toward using the Internet for communication with patients in the future differed significantly (see Table 4). The Internet Advocate (average 4.91) could most easily imagine using the Internet to intensify communication with patients, followed by the Driven Self-expressionist with an average of 4.45. The Efficiency-Oriented physicians took a neutral position, having an average of 3.36. The reason for this might be that the Driven Self-expressionist uses the Internet primarily because of the ease of access to and the vast amount of information, but to a lesser extent for communication with others. The Internet Critic saw almost no reason to use the Internet for communicating with patients (average 2.18). This type uses the Internet for professional activities the least of all types, and results indicate that physicians of this type do not intend to increase use in future. Differences between the user types were significant (*F *= 23.242, *P *< .001).

## Discussion

Whereas academic literature has focused particularly on patients’ use of the Internet for medical content, our survey examined Internet use from the physicians’ perspective. On the basis of physicians’ self-reported behavior, we were able to show that physicians use the Internet for different reasons and that four types of physicians can be identified, based on their motives for professional Internet use. We labeled these physician types (segments) the Internet Advocate, Efficiency-Oriented, Internet Critic, and Driven Self-expressionist. Segments differed with regard to attitudes toward patients who bring health-related Internet-sourced information to a consultation and in their attitudes toward future communication with patients via the Internet. Prescribing behavior did not differ. The results of the survey enabled us to identify physicians’ attitudes toward Internet-informed patients and thus to increase our understanding of physicians’ behavior. The Internet Advocate is open-minded toward the Internet and, for instance, uses social media for professional activities, whereas the Efficiency-Oriented physician primarily uses the Internet because of its efficiency, such as ease of use and saving time when looking for information. The Internet Critic refuses to use the Internet for professional activities, and the Driven Self-expressionist primarily uses the Internet for self-expression. We therefore found that the Internet is used as an information or communication tool in the medical field for different motives. Our investigation revealed that physicians’ willingness to use the Internet for communication with patients in future differed clearly between the physician segments. In particular, Internet Advocates and Driven Self-expressionists could imagine that Internet-based communication will be used more often in future. However, the Efficiency-Oriented and Internet Critic are more reluctant. Finally, if the goal is to prepare physicians for increased Internet use, results suggest that it would be best to address physicians individually according to their established motives for use. Whereas Internet Advocates could be given support in intensifying their use of social media in the medical practice and in providing reliable Internet sources to their patients, Efficiency-Oriented physicians could be informed about further tools to broaden their employment of the Internet for professional activities and become more open to participative Internet use. Driven Self-expressionists are focused on certain Web tools; thus, they could be helped to intensify and extend use of certain Web tools—for instance, to use the Internet for communication rather than just for self-expression. Regarding Internet Critic, it might be important to demonstrate the efficiency and effectiveness of certain Web tools to overcome defensive attitudes. However, given their negative attitude toward the Internet, great efforts may be necessary to change their estimation of the usefulness of the Internet and related applications.

### Study Limitations

Several limitations of this study deserve comment. Our sample was drawn from a physician e-panel, and we conducted the survey by using an online questionnaire. Therefore, only physicians with Internet access and the ability to use the Internet were able to participate. We did not include questions concerning physicians’ Internet literacy in the survey; therefore, we could not consider the possible relations between the physicians’ history of Internet use and user intentions. Given that we used an e-panel, it is possible that physicians with more positive attitudes toward the Internet were overrepresented in the sample.

## Multimedia Appendix 1

Questions and justification of items.

## Multimedia Appendix 2

Summary of the survey.

## Multimedia Appendix 3

Contingency analysis of user segments.

## References

[1] Brooks RG, Menachemi N (2006). Physicians' use of email with patients: factors influencing electronic communication and adherence to best practices. J Med Internet Res.

[2] Hughes B, Joshi I, Lemonde H, Wareham J (2009). Junior physician's use of Web 2.0 for information seeking and medical education: a qualitative study. Int J Med Inform.

[3] Sandars J, Schroter S (2007). Web 2.0 technologies for undergraduate and postgraduate medical education: an online survey. Postgrad Med J.

[4] Iverson Sa, Howard KB, Penney BK (2008). Impact of internet use on health-related behaviors and the patient-physician relationship: a survey-based study and review. J Am Osteopath Assoc.

[5] McGeady D, Kujala J, Ilvonen K (2008). The impact of patient-physician web messaging on healthcare service provision. Int J Med Inform.

[6] Seçkin G (2010). Cyber patients surfing the medical web: Computer-mediated medical knowledge and perceived benefits. Computers in Human Behavior.

[7] Tsai CC, Tsai SH, Zeng-Treitler Q, Liang BA (2007). Patient-centered consumer health social network websites: a pilot study of quality of user-generated health information. AMIA Annu Symp Proc.

[8] Masters K (2008). For what purpose and reasons do doctors use the Internet: a systematic review. Int J Med Inform.

[9] Menachemi N, Prickett C, Brooks R (2011). The use of physician-patient email: a follow-up examination of adoption and best-practice adherence 2005-2008. J Med Internet Res.

[10] Ortega Egea JM, González MV, Menéndez MR (2010). eHealth usage patterns of European general practitioners: a five-year (2002-2007) comparative study. Int J Med Inform.

[11] Schiffman L, Kanuk L (2007). Consumer Behavior. 9th edition.

[12] Solomon MR (2007). Consumer Behavior: Buying, Selling and Being. 7th edition.

[13] Sechrest R (2010). The internet and the physician-patient relationship. Clin Orthop Relat Res.

[14] Jo HS, Hwang M-S, Lee H (2010). Market segmentation of health information use on the Internet in Korea. Int J Med Inform.

[15] Rice R (2006). Influences, usage, and outcomes of Internet health information searching: multivariate results from the Pew surveys. Int J Med Inform.

[16] Bennett N, Casebeer L, Zheng S, Kristofco R (2006). Information-seeking behaviors and reflective practice. J Contin Educ Health Prof.

[17] (2007). Manhattan Research LLC.

[18] Umefjord G, Sandström H, Malker H, Petersson G (2008). Medical text-based consultations on the Internet: a 4-year study. Int J Med Inform.

[19] Wald HS, Dube CE, Anthony DC (2007). Untangling the Web--the impact of Internet use on health care and the physician-patient relationship. Patient Educ Couns.

[20] McKinlay J, Marceau L (2008). When there is no doctor: reasons for the disappearance of primary care physicians in the US during the early 21st century. Soc Sci Med.

[21] Sommerhalder K, Abraham A, Zufferey MC, Barth J, Abel T (2009). Internet information and medical consultations: experiences from patients' and physicians' perspectives. Patient Educ Couns.

[22] Buetow S, Jutel A, Hoare K (2009). Shrinking social space in the doctor-modern patient relationship: a review of forces for, and implications of, homologisation. Patient Educ Couns.

[23] Edwards M, Davies M, Edwards A (2009). What are the external influences on information exchange and shared decision-making in healthcare consultations: a meta-synthesis of the literature. Patient Educ Couns.

[24] Giveon S, Yaphe J, Hekselman I, Mahamid S, Hermoni D (2009). The e-patient: a survey of israeli primary care physicians' responses to patients' use of online information during the consultation. Isr Med Assoc J.

[25] Gray NJ, Klein JD, Noyce PR, Sesselberg TS, Cantrill JA (2005). Health information-seeking behaviour in adolescence: the place of the internet. Soc Sci Med.

[26] Ahmad F, Hudak LP, Bercovitz K, Hollenberg E, Levinson W (2006). Are physicians ready for patients with Internet-based health information?. J Med Internet Res.

[27] Rhebergen MDF, Lenderink AF, van Dijk FJ, Hulshof CT (2012). Comparing the use of an online expert health network against common information sources to answer health questions. J Med Internet Res.

[28] Sillence E, Briggs P, Harris PR, Fishwick L (2007). How do patients evaluate and make use of online health information?. Soc Sci Med.

[29] Anderson JG, Rainey MR, Eysenbach G (2003). The impact of CyberHealthcare on the physician-patient relationship. J Med Syst.

[30] McMullan M (2006). Patients using the Internet to obtain health information: how this affects the patient-health professional relationship. Patient Educ Couns.

[31] Murray E, Pollack L, White M, Lo B (2007). Clinical decision-making: physicians' preferences and experiences. BMC Fam Pract.

[32] Hart A, Henwood F, Wyatt S (2004). The role of the Internet in patient-practitioner relationships: findings from a qualitative research study. J Med Internet Res.

[33] Theodorou M, Tsiantou V, Pavlakis A, Maniadakis N, Fragoulakis V, Pavi E, Kyriopoulos J (2009). Factors influencing prescribing behaviour of physicians in Greece and Cyprus: results from a questionnaire based survey. BMC Health Serv Res.

[34] Richard C, Lussier MT (2007). Measuring patient and physician participation in exchanges on medications: Dialogue Ratio, Preponderance of Initiative, and Dialogical Roles. Patient Educ Couns.

[35] Bartlett YK, Coulson NS (2011). An investigation into the empowerment effects of using online support groups and how this affects health professional/patient communication. Patient Educ Couns.

[36] Bosslet GT, Torke AM, Hickman SE, Terry CL, Helft PR (2011). The patient-doctor relationship and online social networks: results of a national survey. J Gen Intern Med.

[37] Bergmo TS, Kummervold P, Gammon D, Dahl LB (2005). Electronic patient-provider communication: will it offset office visits and telephone consultations in primary care?. Int J Med Inform.

[38] Patt RM, Houston KT, Jenckes WM, Sands ZD, Ford ED (2003). Doctors who are using e-mail with their patients: a qualitative exploration. J Med Internet Res.

[39] Agha Z, Roter LD, Schapira MR (2009). An evaluation of patient-physician communication style during telemedicine consultations. J Med Internet Res.

[40] Santana S, Lausen B, Bujnowska-Fedak M, Chronaki C, Kummervold PE, Rasmussen J, Sorensen T (2010). Online communication between doctors and patients in Europe: status and perspectives. J Med Internet Res.

[41] Theodoridis S, Koutroumbas K (2008). Pattern Recognition. 4th edition.

[42] Raab A, Poost A, Eichhorn S (2009). Marketingforschung: Ein Praxisorientierter Leitfaden.

[43] Lewis PJ, Tully MP (2011). The discomfort caused by patient pressure on the prescribing decisions of hospital prescribers. Res Social Adm Pharm.

[44] Hair JF, Black B, Babin B, Anderson RE, Tatham RL (2006). Multivariate Data Analysis. 6th edition.

