# Supplementary material for: Physicians' Motives for Professional Internet Use and Differences in Attitudes Toward the Internet-Informed Patient, Physician–Patient Communication, and Prescribing Behavior
Source: Med 2 0. 2012 Jul 6;1(2):e2. doi: 10.2196/med20.1996 (PMC4084769; doi:10.2196/med20.1996)
Supplement: Supplementary file 1 [file med20_v1i2e2_app1.pdf]

Appendix 2 – Questions and Justification of Items

1. What feelings do you have towards the Internet and web 2.0 in general?

Very negative						Very positive	No reply	Adopted from
O	O	O	O	O	O	O	O	Edit through medical expert advice

2. The following questions deal with Internet use for private and professional activities.

a) If you think of your own Internet use, <u>how many hours</u> do you use the Internet for <u>private</u> activities on average?
(Please choose one of the response options (per day <u>or</u> per week <u>or</u> per month) that best describe your own use.)

Average use of the Internet **per day** __________
Average use of the Internet **per week** __________
Average use of the Internet **per month** __________

b) If you think of your own Internet use, <u>how many hours</u> do you use the Internet for <u>professional</u> activities on average?
(Please choose one of the response options (per day <u>or</u> per week <u>or</u> per month) that best describe your own use.)

Average use of the Internet **per day** __________
Average use of the Internet **per week** __________
Average use of the Internet **per month** __________

3. Could you imagine using the Internet for communication with your patients more often in the future?

I absolutely cannot imagine						I can easily imagine	No reply	Adopted from
O	O	O	O	O	O	O	O	[18]

4. Please tell us your motives for your Internet use for professional activities?

**I use the Internet, because …**	I absolutely disagree						I absolutely agree	No reply	Adopted from
… I can look for information easily.	O	O	O	O	O	O	O	O	[2,13]
… it offers a vast amount of information.	O	O	O	O	O	O	O	O	[2]
… it offers current information.	O	O	O	O	O	O	O	O	[14,16]
… it offers different formats, e.g. social networks, podcasts or health bulletin boards.	O	O	O	O	O	O	O	O	[2,29]
… the information is easy to understand.	O	O	O	O	O	O	O	O	[2]
… I want to save time.	O	O	O	O	O	O	O	O	[13]
… I can get connected with others easily.	O	O	O	O	O	O	O	O	[16]
… I want to get familiar with the information sources of my patients.	O	O	O	O	O	O	O	O	Edit through medical expert advice

… I want to keep up with other physicians.	O	O	O	O	O	O	O	O	[17]
… It offers an opportunity to express oneself.	O	O	O	O	O	O	O	O	[2,17]
… it is important to be on the web as a physician.	O	O	O	O	O	O	O	O	[2]
… I want to be on the cutting-edge.	O	O	O	O	O	O	O	O	Edit through medical expert advice
… I prefer to inform myself anonymously.	O	O	O	O	O	O	O	O	[29]
… it can be used 24/7.	O	O	O	O	O	O	O	O	[29]

5. Please indicate the degree to which you agree with the statements below.

**If a patient brought some health-related information to consultation …**	I absolutely disagree						I absolutely agree	No reply	Adopted from
… I think it is generally positive.	O	O	O	O	O	O	O	O	[21,24]
… I am prepared to correct wrong, incomplete and misunderstood information.	O	O	O	O	O	O	O	O	[21]
… I sometimes feel I might lose authority and control.	O	O	O	O	O	O	O	O	[26]
… I expect a more time-consuming patient visit than with uninformed patients.	O	O	O	O	O	O	O	O	[22,29]
… the physician-patient relationship will be improved by better communication.	O	O	O	O	O	O	O	O	[4,20,21]
… I would be more likely to prescribe a desired medication than if the patient was uninformed.	O	O	O	O	O	O	O	O	[31,33, 43]

Demographic Information:

Sex: [1] male
[2] female

Year of Birth: __________

Medical Field:

[01] General Practitioner
[02] Dermatologist
[03] Orthopedist
[99] Other: ________________________

You normally treat patients with:

a) chronic diseases ( )
b) acute diseases ( )
c) both acute and chronic diseases ( )
