# Supplementary material for: Physicians' Motives for Professional Internet Use and Differences in Attitudes Toward the Internet-Informed Patient, Physician–Patient Communication, and Prescribing Behavior
Source: Med 2 0. 2012 Jul 6;1(2):e2. doi: 10.2196/med20.1996 (PMC4084769; doi:10.2196/med20.1996)
Supplement: Supplementary file 2 [file med20_v1i2e2_app2.pdf]

Appendix 1 – Summary of Survey

Category	*Checklist Item*	*Explanation*
**Design**		
	**Target population and sample frame**	German physicians from three medical fields including general practitioners, orthopedists and dermatologists.<br>The sample frame was determined by GfK HealthCare in order to offer a representative status quo of physicians, as well as to have legitimation and opportunity of statistical analysis. The sample size was determined as follows:<br>General practitioners: n = 125<br>Orthopedists: n = 80<br>Dermatologists: n = 80<br>Physicians polled are members of GfK's physicians e-panel, and were invited for participation based on their medical field.
**Informed consent process**		
	**Informed consent**	Selected physicians received an invitation mail to participate in our survey in which they were informed about the duration, the content of the questionnaire as dealing with physicians' current Internet use, their communication with their patients and their appreciation of future Internet use. Additional information was given in the introduction part of the online questionnaire. Further, they were referred to the cooperation with the University of Klagenfurt.
	**Data protection**	Data were protected by the GfK HealthCare. Physicians were informed that e-mail addresses and user IDs were stored separately from their responses in the survey.
**Development and pre-testing**		
	**Development**	The final questions of the survey were determined in cooperation with two medical experts from the GfK HealthCare. The transformation of the questionnaire into an online format was made by the research company.
	**Pre-Testing**	A pre-test with three physicians was conducted by the GfK HealthCare. Comments on the usability of the online survey and the comprehensibility of the content were considered and implemented.
**Recruitment process and description of the sample having access to the questionnaire**		
	**Closed survey**	Only physicians who are members of the GfK e-panel were able to participate in the survey.

	**Contact mode**	Selected participants received an invitation e-mail to participate in the closed survey. The e-mail contained a link to the online survey.
**Survey Administration**		
	**Incentives**	Monetary incentives were offered to the participants who had completed the online questionnaire. Incentive amount was based on the common ADM-guidelines (ADM: Arbeitskreis Deutscher Markt- und Sozialforschungs-institute).
	**Context of Survey**	Since the survey dealt with physicians' Internet use, results of our study only contain responses from physicians who use the Internet regularly and have Internet access.
	**Time of Data Collection**	Data were collected from 13th of December 2010 to 14th of January 2011.
	**Number of Items**	Number of items per screen dependent on the questions. But there were a maximum of 12 statements on one site.
	**Number of screens**	20 screens
	**Completeness Check**	All items contained a non-response option, such as "no answer" otherwise it was not possible to move on to the next question.
	**Review Step**	A back button was given to review and change answers.
	**Response Rate**	64% (Ratio between the completed questionnaires and the emails sent.)
**Prevention multiple entries from the same individual**		
	**Link used**	Registration – membership to the e-panel is necessary; the link to the survey is only accessible once. Every respondent received a unique link that enables access the survey once. Hence, every activated link is counted only one time.
**Analysis**		
	**Handling of incomplete questionnaires**	In our study, only completed questionnaires were used.
