# Supplementary material for: Physicians' Motives for Professional Internet Use and Differences in Attitudes Toward the Internet-Informed Patient, Physician–Patient Communication, and Prescribing Behavior
Source: Med 2 0. 2012 Jul 6;1(2):e2. doi: 10.2196/med20.1996 (PMC4084769; doi:10.2196/med20.1996)
Supplement: Supplementary file 3 [file med20_v1i2e2_app3.pdf]

Appendix 3 – Contingency Analysis of User Segments

Motives for using the Internet for professional activities	Internet Advocate			Efficiency-Oriented			Internet Critic			Driven Self-Expressionist			F	*P*
	%[a]	n[a]	mean	%[a]	n[a]	mean	%[a]	n[a]	mean	%[a]	n[a]	mean		
Factor 1: Cutting-edge and Self-expression														
It is important to be on the web as a physician.	87	88	5.79	9	8	2.56	28	8	3.24	84	43	5.76	128.354	<.001
It offers an opportunity to express oneself.	71	72	5.39	5	5	2.12	14	4	2.90	69	35	5.18	110.206	<.001
I want to be on the cutting-edge.	82	83	5.74	14	13	2.86	17	5	3.07	69	35	5.25	71.296	<.001
I want to keep up with other physicians.	61	62	4.88	2	2	2.12	17	5	2.69	35	18	3.84	59.354	<.001
Factor 2: Efficiency and Effectiveness														
I can look for information easily.	100	101	6.64	100	93	6.72	45	13	4.21	100	51	6.80	115.694	<.001
It offers a vast amount of information.	100	101	6.63	99	92	6.63	52	15	4.45	100	51	6.76	90.220	<.001
It offers current information.	99	100	6.37	91	85	6.04	31	9	4.03	92	47	5.69	45.154	<.001
I want to save time.	96	97	6.35	84	78	5.78	45	13	4.34	96	49	6.47	27.032	<.001
Factor 3: Diversity and Convenience														
The information is easy to understand.	90	91	5.78	43	40	4.35	14	4	3.38	22	11	3.59	53.125	<.001
It offers different formats, e.g. Social Networks, podcasts or health bulletin boards.	87	88	5.82	42	39	4.05	14	4	2.90	26	13	3.37	45.932	<.001

[a] (range from 5, I agree to 7, I absolutely agree)
